# Supplementary material for: Gigantic jet discharges evolve stepwise through the middle atmosphere
Source: Nat Commun. 2019 Sep 25;10:4350. doi: 10.1038/s41467-019-12261-y (PMC6761152; doi:10.1038/s41467-019-12261-y)
Supplement: Supplementary file 1 — Supplementary Information [file 41467_2019_12261_MOESM1_ESM.pdf]

## Supplementary information

# Gigantic jet discharges evolve stepwise through the middle atmosphere

*van der Velde et al.*

## Supplementary Figures

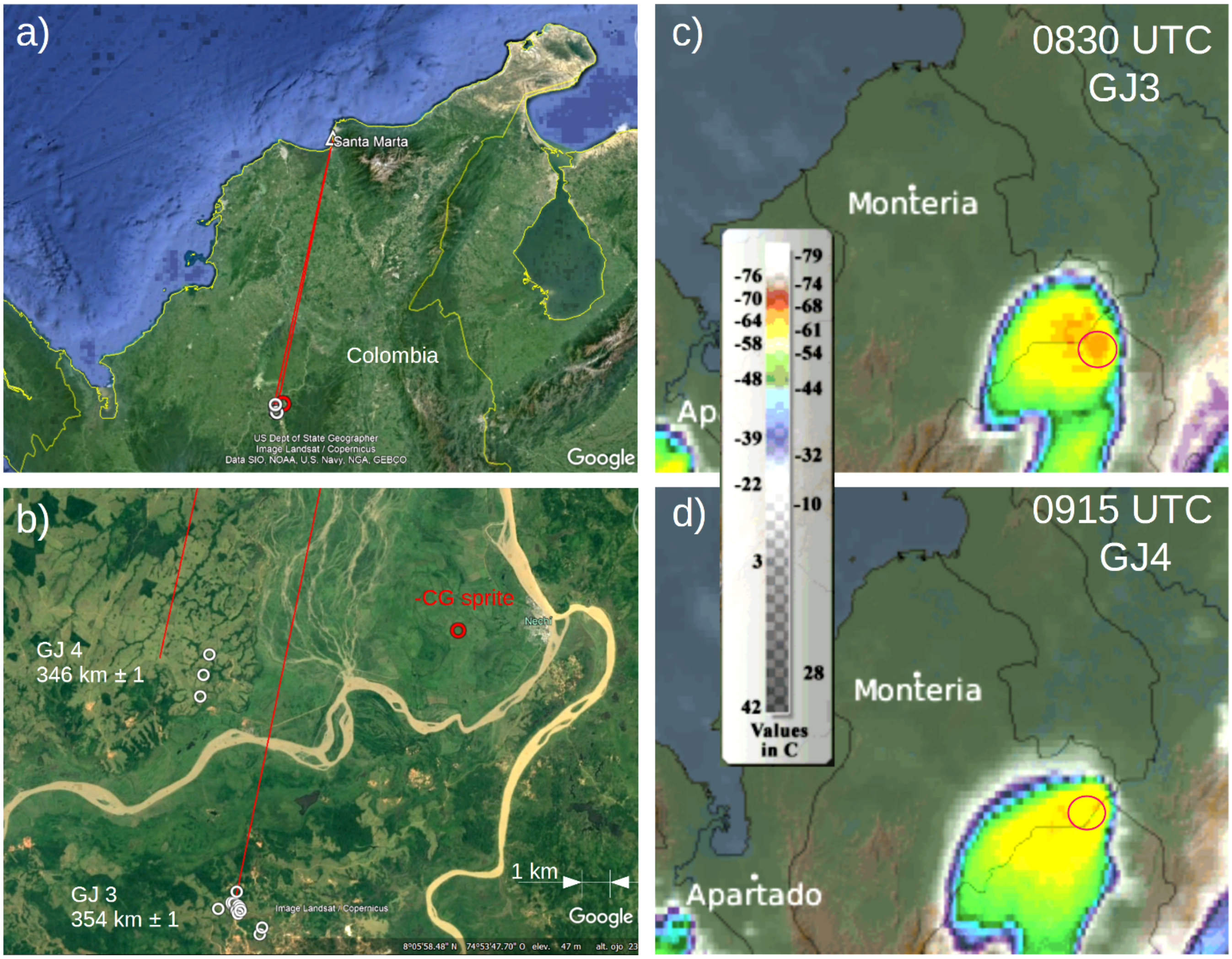


**Supplementary Figure 1 | Geographic location of the two gigantic jets of 14 August 2017.**

(a) and (b) Great circle paths from the observation site near Santa Marta airport to the gigantic jets, determined from star backgrounds. The Keraunos (LINET) total lightning detections corresponding to event GJ 3 are plotted as white circles. (c) and (d) show the GOES satellite cloud top temperatures and the location in the circle of GJ 3 and 4 (source: Aeronáutica Civil, Colombia).

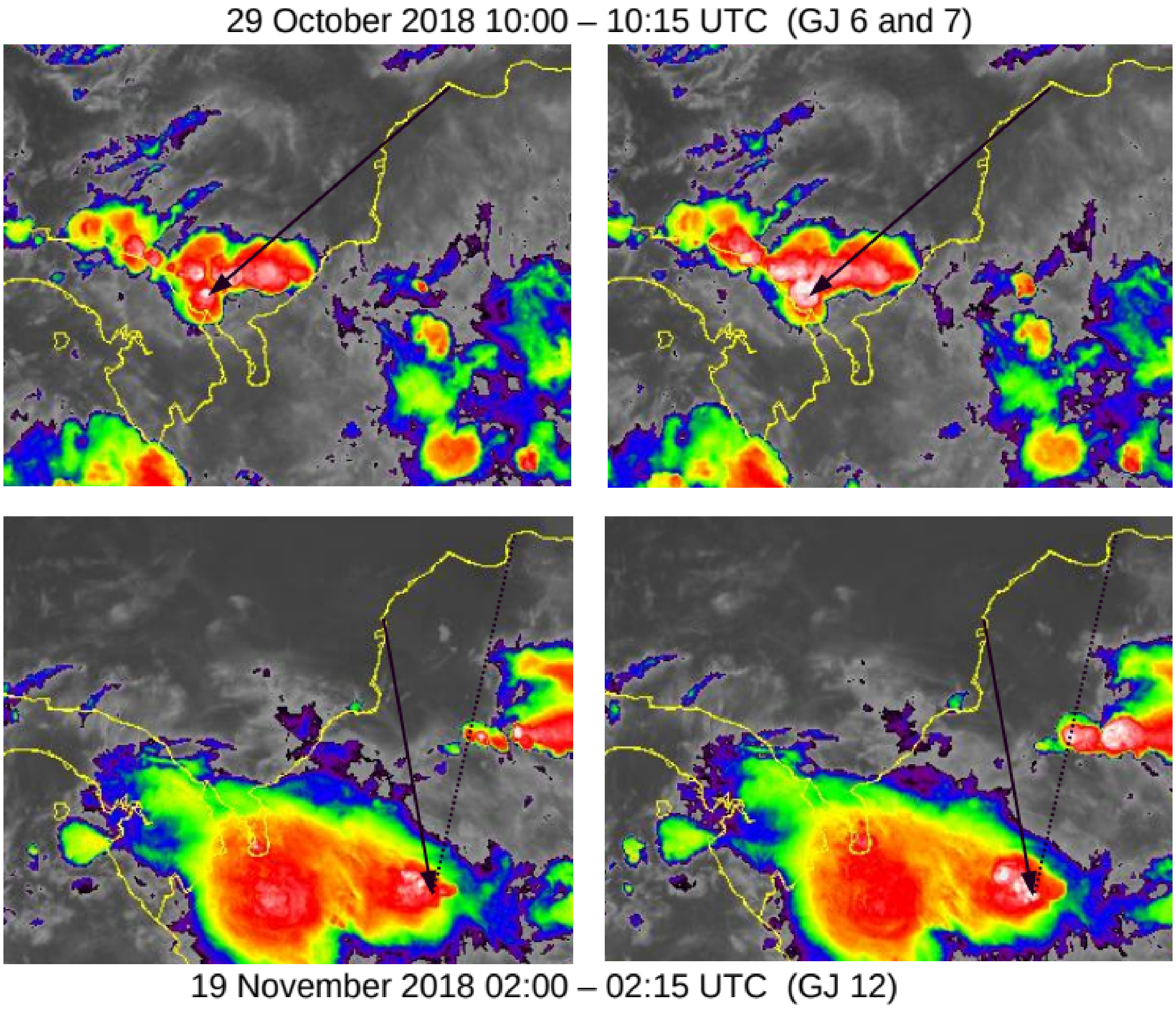


**Supplementary Figure 2 | Infrared satellite images of two thunderstorms producing gigantic jets.** The cases of 29 October (10:09:55 and 10:10:44 UTC) observed from Barranquilla (top), and 19 November 2018 (02:13:39 UTC) observed from Cartagena and Santa Marta (bottom). In both cases the growing overshooting tops can be identified (in white). The locations were marked also by Keraunos lightning detections and Geostationary Lightning Mapper (GLM) (not shown). Image source: NASA Marshall Space Flight Center, USA.

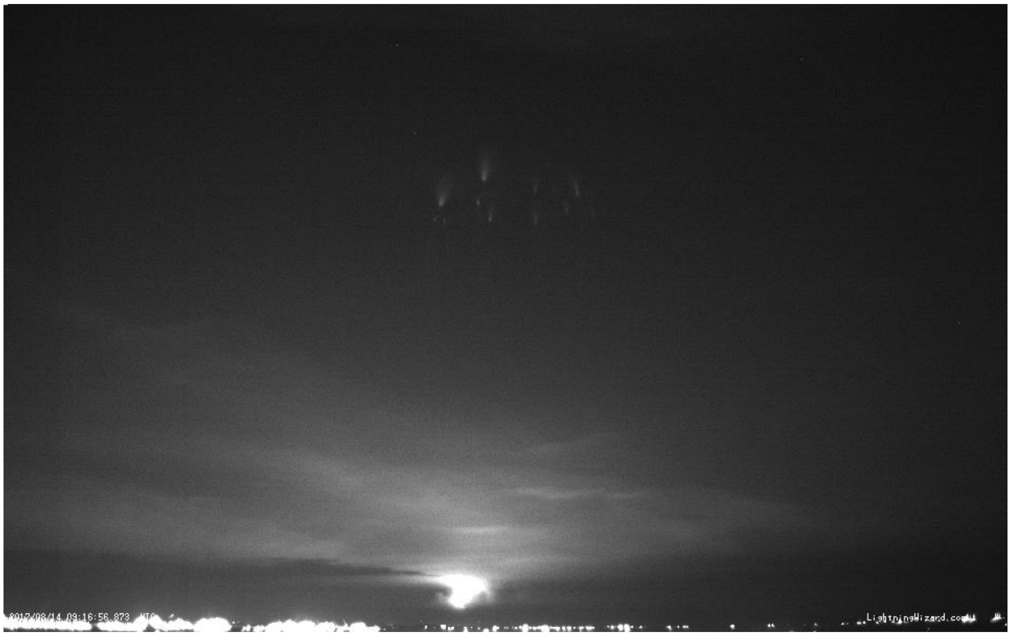

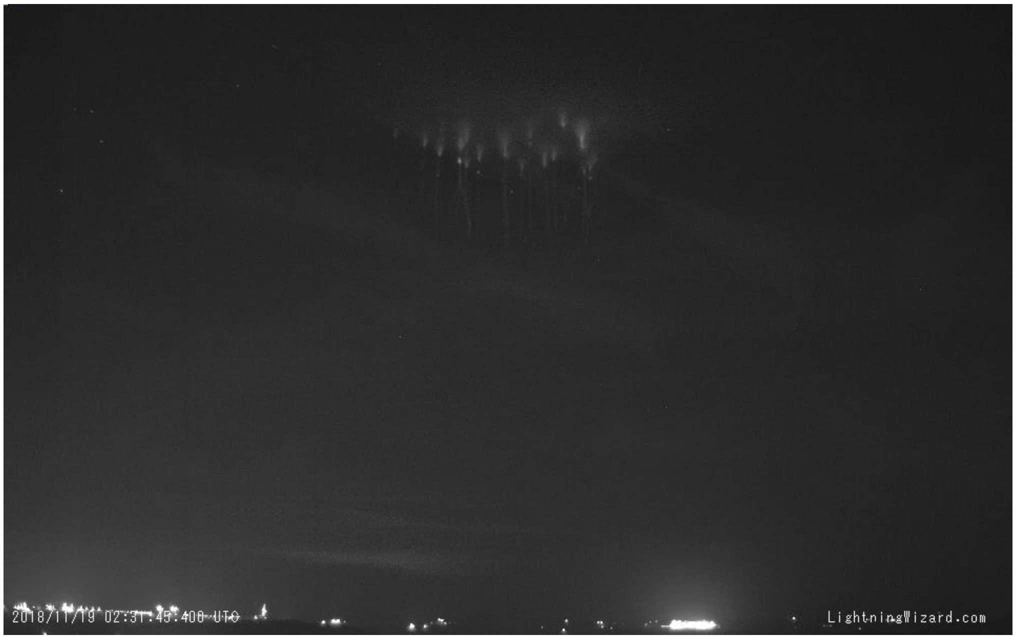


**Supplementary Figure 3 | Negative sprites surrounding the times of occurrence of GJ 4 and 12.**

(top) Sprite triggered by a -82 kA negative polarity cloud-to-ground stroke, 2 minutes after GJ 4, 14 August 2017 at 09:16:56.937 UTC. (bottom) Sprite triggered by a -112 kA negative polarity cloud-to-ground stroke, 18 minutes after GJ 12, 19 November 2018 at 02:31:45.465 UTC.

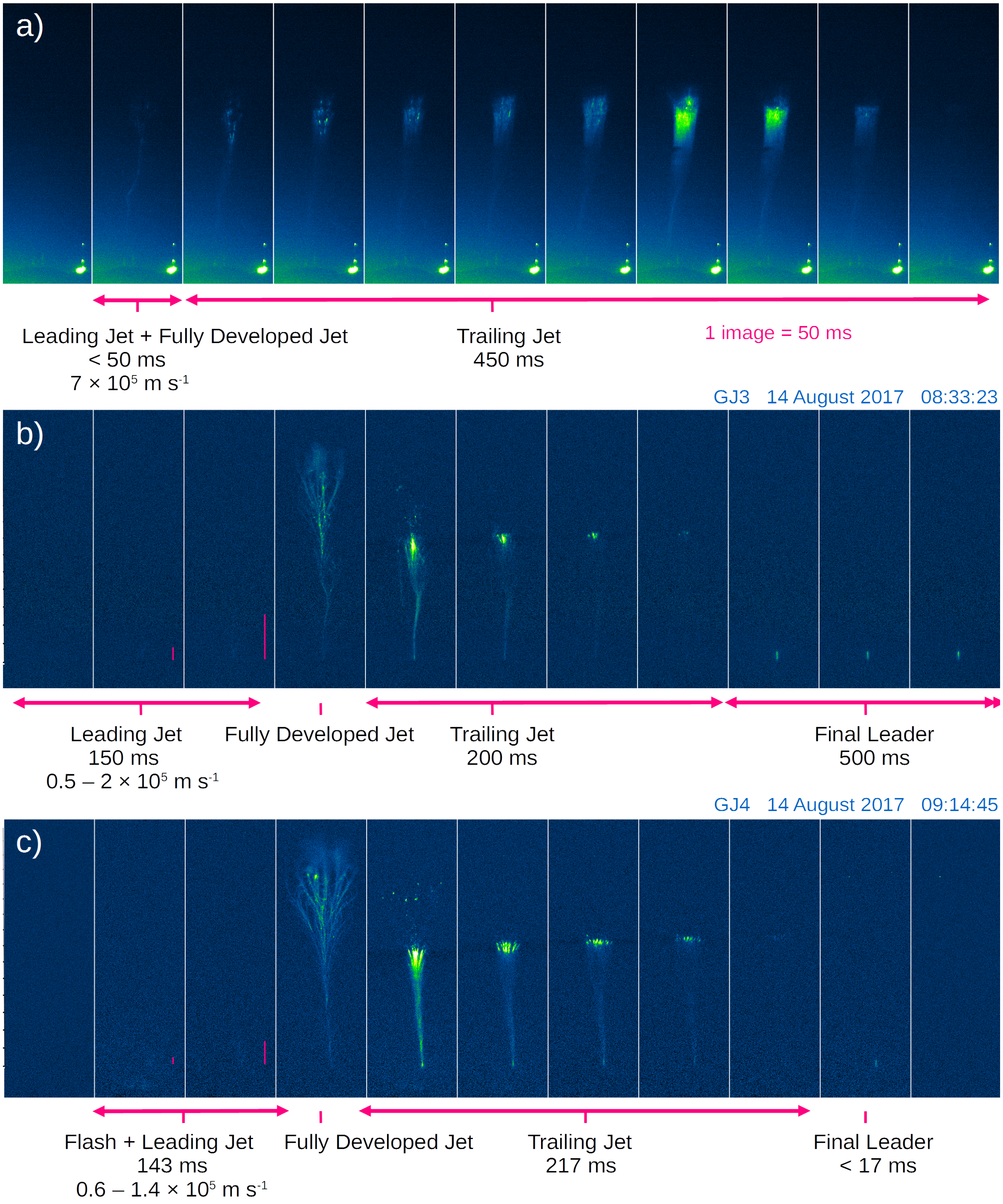


**Supplementary Figure 4 | High resolution sequences of gigantic jet evolution (2017) and their evolution stages.**

GJ 2 (a) is an unusual event. The channel forms a few beads at the top, widens, and suddenly brightens with some similarity to a Trailing Jet, without rising beads. The initial speed appears to be faster than the other events. GJ 3 (b) shows a slower, very faint Leading Jet (indicated by a vertical magenta bar) and a long-lasting Final Leader. GJ 4 (c), here reduced in frame rate to match the others. Full data are available as indicated in the Data Availability statement.

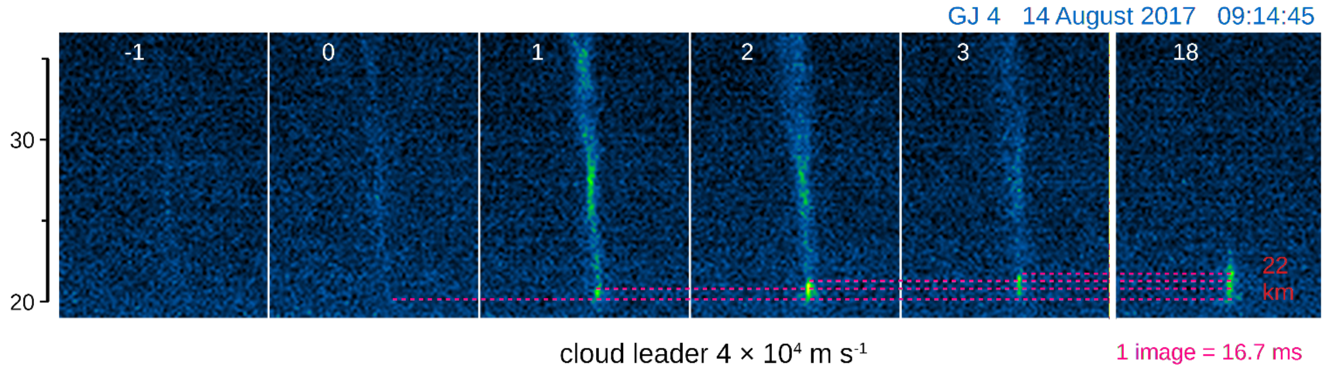


**Supplementary Figure 5 | Zoomed in sequence of the lower jet and cloud leader in GJ 4.**

The cloud leader (bright segment indicated by magenta dashed lines) was still hidden during leading jet and and fully developed jet stages, appeared during the trailing jet stage, extending progressively upwards. Frame numbers are indicated on top. The final frame 18 (283-300 ms after the fully developed jet, frame 0) is an isolated rebrightening of the cloud leader. The intensified Miro high-speed camera also captured the cloud leader, but could not resolve the limited upward movement. The rebrightening lasted less than 1 frame (<1.1 ms) in that video.

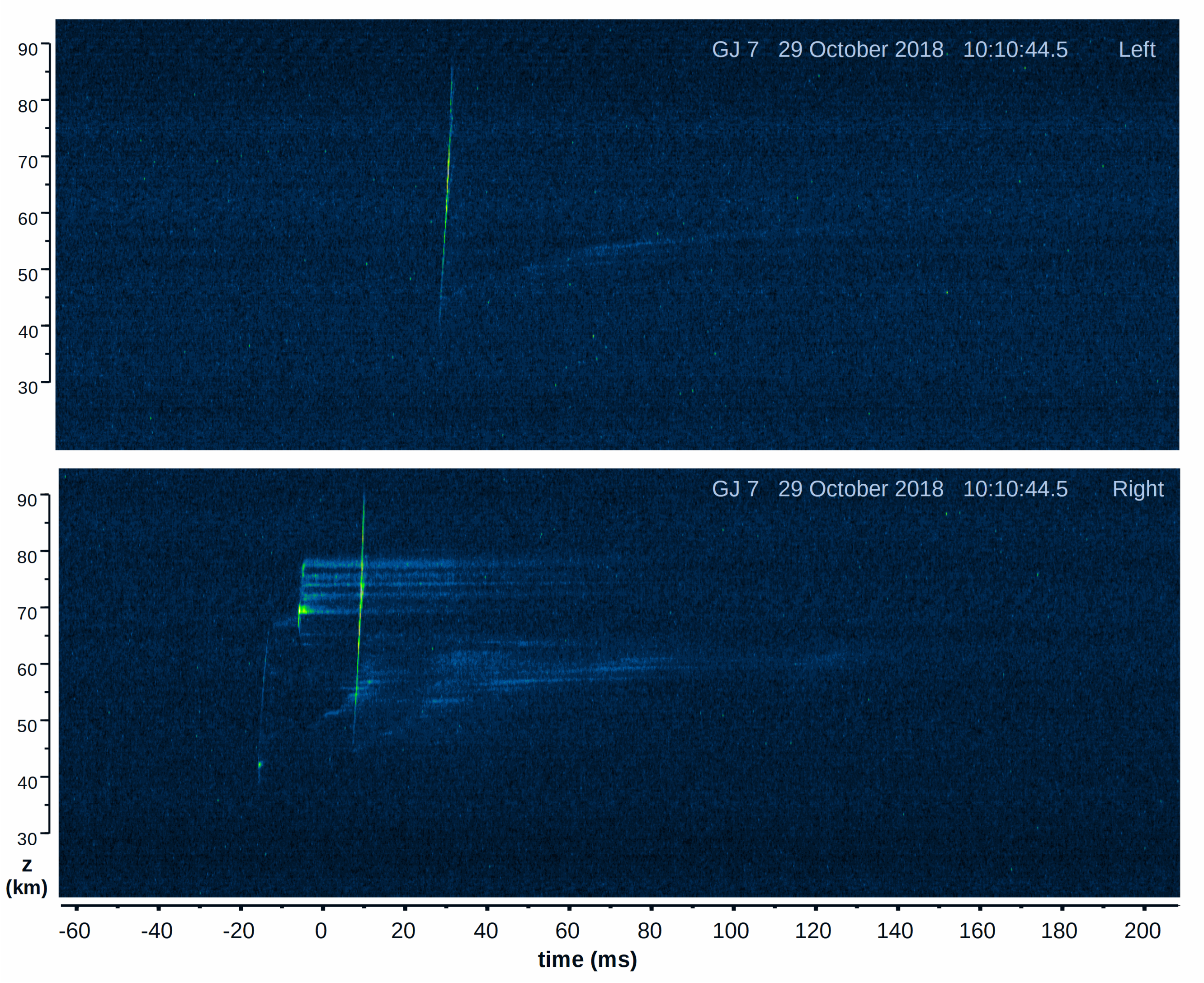


**Supplementary Figure 6 | Time-altitude-luminosity graphs of GJ 7 separate branches.**

These separated plots show better how the glow of the upper beads at the right branch suddenly decreases (at t=30 ms) as the left branch completes development. The time scale and aspect ratio (slopes) are identical to the combined image in the main article. Note that the transient noisy speckles of the intensified images have not been filtered out here.

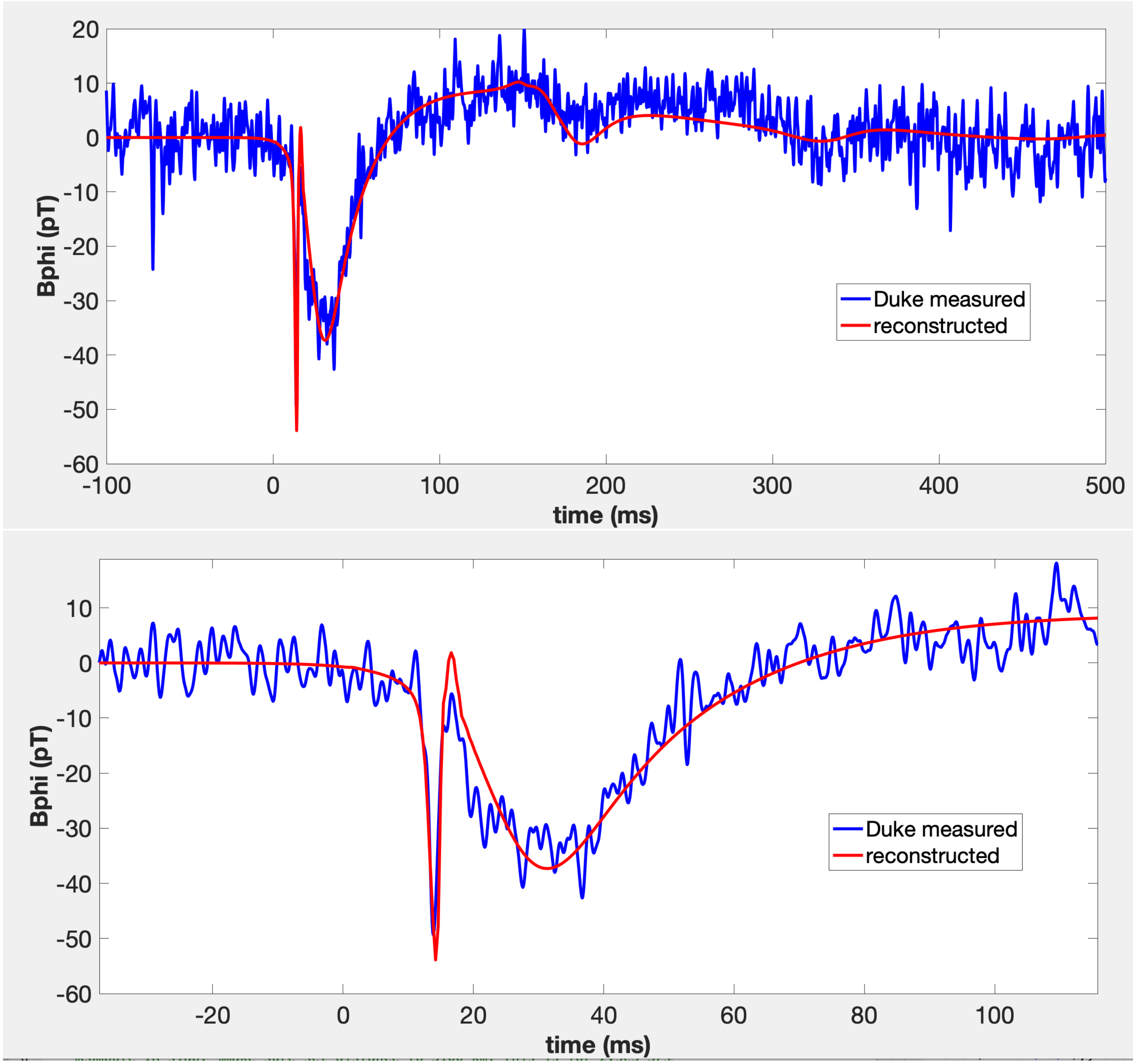


**Supplementary Figure 7 | The fit between the measured and reconstructed radio signal at Duke University.**

The reconstructed waveform is used for the extracted current moment for GJ 12. Bottom: zoomed in.

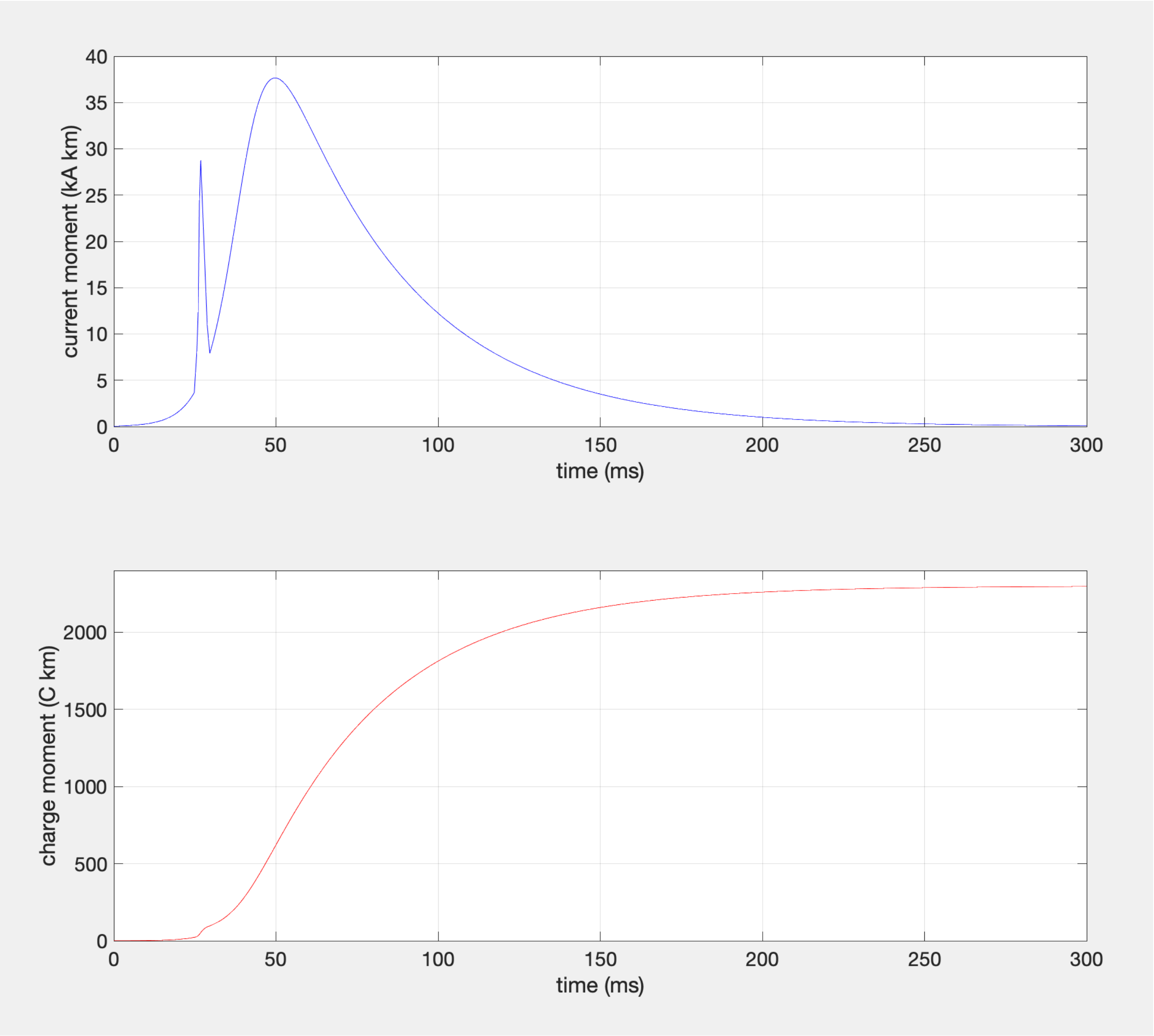


**Supplementary Figure 8 | Current moment waveform and charge moment change for GJ 12.**

From the Duke University measurement. Top: Evolution of current moment. Bottom: Accumulated charge moment.

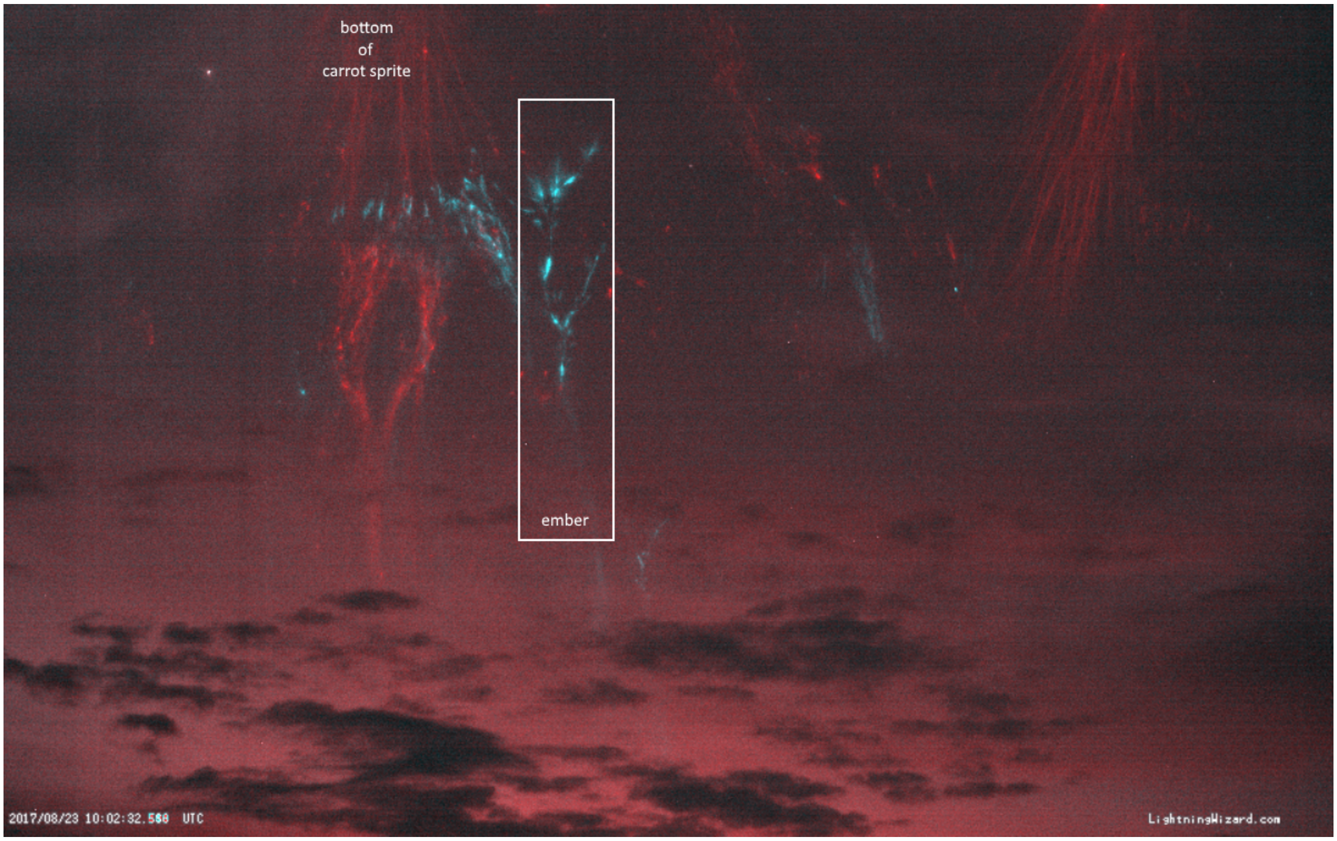


## Supplementary Figure 9 | Large sprite and a secondary upward discharge.

A group of carrot sprites (in red, top filaments) and the secondary transient luminous event of negative polarity known as “ember” (in cyan), growing towards the lower positive streamers of carrot sprites (top). The angle of view is 25.3° by 15.8°. The white box highlights the area used for the fast camera sequence in Supplementary Fig. 10. This camera also captured the low altitude parts of the ember below the field of view of the fast camera, which used a narrower 50mm lens than the ones used for the 2017 gigantic jets. To the left of the ember is another type of secondary upward discharge retracing the channels left by an earlier sprite.

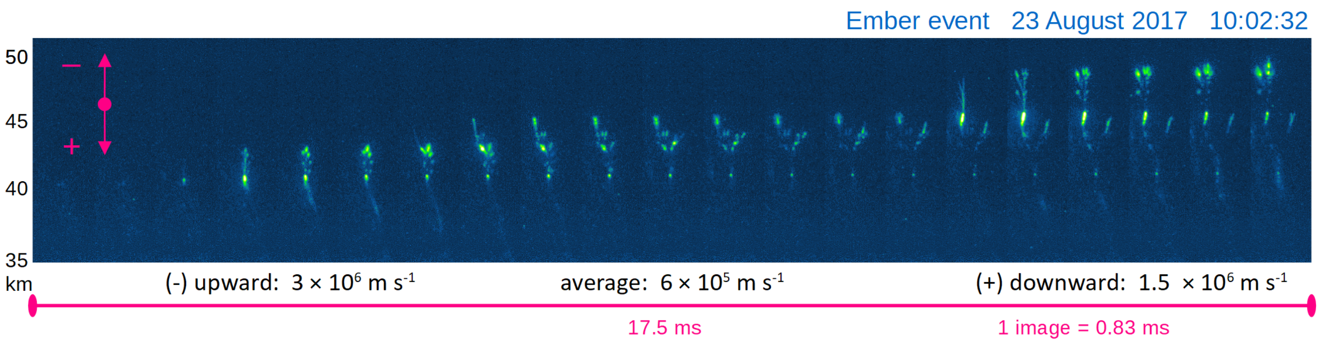


## Supplementary Figure 10 | Evolution of an ember discharge.

Fast camera image sequence (1200 frames per second) of the ember event highlighted in Supplementary Fig. 9, occurring under positive streamers of carrot sprites. At the start of this sequence a barely detectable trace is already present between 40 and 35 km altitude. The average upward propagation which a slow camera may observe is significantly slower than the steps themselves, as development is paused after each step. Note the downward positive streamers retracing the older trajectory.

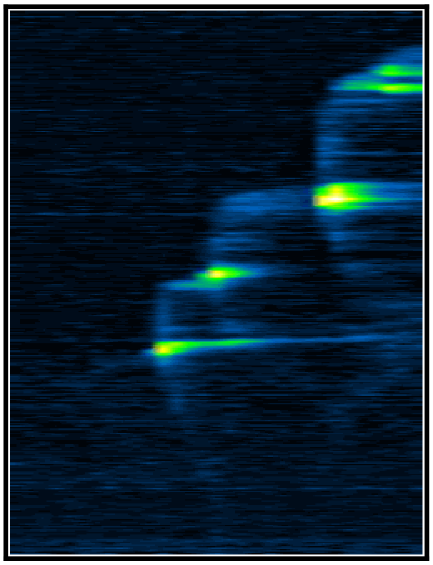

## Supplementary Figure 11 | Time-altitude-luminosity graph of the ember.

The vertical (35-50 km) and horizontal axes (17.5 ms) correspond to the image sequence of Supplementary Fig. 10. This graph shows more clearly the repeated upward and downward luminosity movements in the discharge.

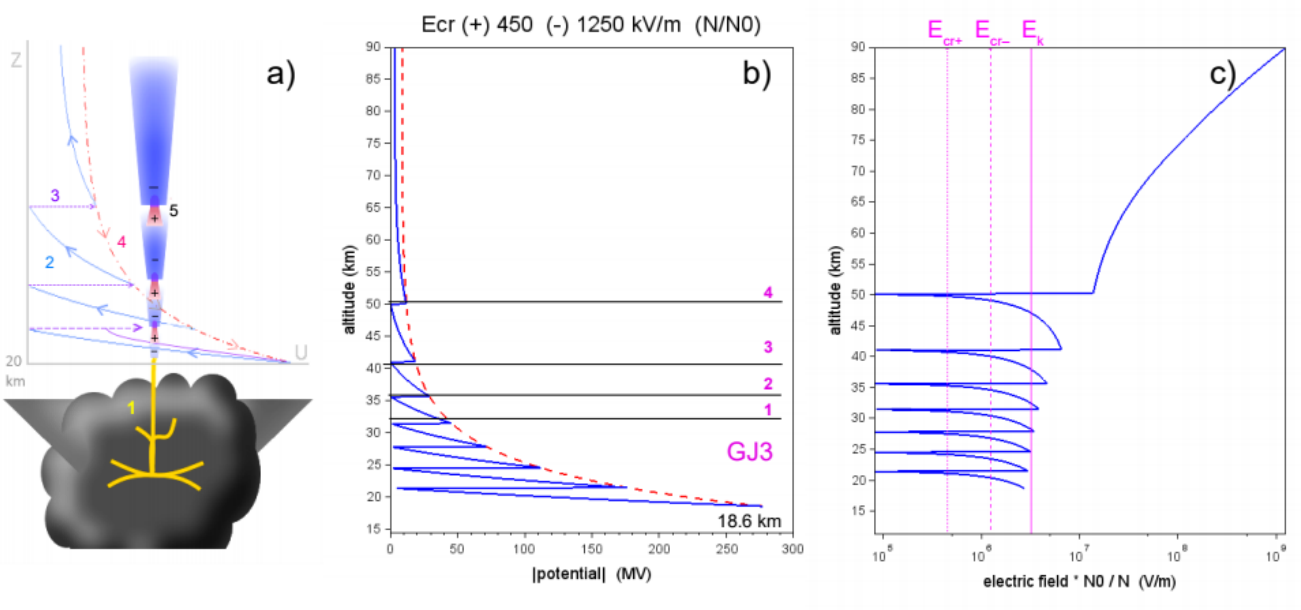


**Supplementary Figure 12 | Schematic of the steps in a gigantic jet and electrostatic model.**

**(a)** Schematic of the gigantic jet, with numbered steps described in the Supplementary Discussion. **(b)** Evolution of potential at the tip of the discharge as it moves up. The altitudes of observed steps in event GJ 3 are matched within 1 km assuming a leader tip with potential of 277 MV at 18.6 km, with commonly used stability fields for positive and negative streamer corona. **(c)** The electric field 100 m in front of streamer corona, assumed to have a radius of 10 m. Vertical lines indicate the threshold fields for streamer propagation $E_{cr+}$ and $E_{cr-}$ and the breakdown threshold $E_k$. The code of the electrostatic model is available from the authors.

# Supplementary Discussion

## Ember – a secondary discharge under sprites

During the 2017 campaign a type of discharge similar to jets was recorded, offering a detailed view of processes that may occur during the steps in gigantic jets. The event was a form of "secondary jet" [1] growing upwards during a sequence of large carrot sprites (**Supplementary Fig. 9)**. It occurred over northeastern Colombia on 23 August 2017, 10:02:32 UTC. These secondary discharges involve negative streamers growing upward from low altitudes (25-40 km) toward the positive charge deposited by sprite streamers[2]. It has never been confirmed that these secondary phenomena indeed grow upwards from the cloud. Secondary transient luminous events come in various morphologies[3,4] Here we discuss one form with an evolution (**Supplementary Fig. 10**) previously described as "embers" [4].

This event did not follow any trajectory previously conditioned by sprite streamers, which is why we believe it to be similar to the processes during the leading jet stage in a gigantic jet, under a driving electric field small enough to prevent unlimited negative streamer growth towards the ionosphere. The proportions of a preceding carrot sprite[5] allowed a rough estimate of the altitude scale, as some part of the ember appeared to connect sidewards to it. The corresponding estimated distance is 120 km, which is within the perimeter of the thundercloud determined from satellite images.

**Supplementary Fig. 10** shows initial development from a weak bead with a barely detectable luminous channel underneath, which would be a streamer zone of unknown origin at altitudes dominated by blue emissions. **Supplementary Fig. 11** shows the corresponding time-altitude-luminosity plot. The weak bead brightens over ~2 ms while emitting a new upward negative streamer. At the same time, a positive streamer is seen to propagate downward along the previous channel at $1.5 \times 10^{6}$ m s$^{-1}$. New beads form almost immediately at the new upper extremities of the negative streamers. After 3-4 ms, one of the beads intensifies and a new streamer steps upward. This repeats again with a brighter bead and longer negative streamer 8 ms later, the tips turning into beads, while positive streamer luminosity is again clearly visible in the lower section, under the location of the bead that started the sequence. The channel luminosity then increases again between the initial and second bead, showing how the different sections still communicate with each other. The average upward

propagation speed derived from the slope of overall development is about $6 \times 10^5$ m $s^{-1}$ (9 km in ~15 ms). The step velocity is ~ $3 \times 10^6$ m $s^{-1}$. These values are comparable to those in GJ 3.

This sequence can be interpreted as the largest scale of pilot system propagation observed in nature. The difference with a negative leader is clear: here the beads (stems) do not extend bidirectionally and then connect to a main leader. The action of backward positive streamers increases the flow of electrons in the upward direction along the previously formed channels, renewing the potential gradient to launch new negative streamers. A simple concept based on Gallimberti et al.[6] , Raizer et al.[7,8] and Da Silva and Pasko[9] is applied to the gigantic jet case in the following Supplementary Discussion section.

Comparing this event to the recorded gigantic jet steps, there are some interesting similarities and differences. (1) The luminosity in the ember event is maintained almost continuously. This may be a result of its higher altitude than the lower half of the gigantic jet, where red light is not quenched. (2) The bright persisting beads in the ember. One bead was detected in GJ 7 at the onset of the final jump, which started at 41 km altitude, perhaps also in GJ 12 (weaker, 38 km altitude), but none in the observed steps below 40 km. However, one can note the presence of small beads in the LJ stage in several events of Soula et al.[10] and Liu et al.[11,12] (3) The lower positive streamers appear slower in the ember event and can be traced down to lower altitudes. (4) The beads in the ember persist while slowly moving upward. This reminds of the 1-2 × $10^4$ m $s^{-1}$ beads in the transition zone of the trailing jet which occur in the same altitude range. (5) An upward moving luminosity wave can be seen in the lower ember channel, late in the event. Such low altitude dynamics will likely become detectable when gigantic jets are recorded at similar distances as this ember.

**Electrostatic model with stepping in the negative streamer zone**

Let us now assume the steps in the upward propagation in gigantic jets are not produced by a stepped leader, but by a negative streamer corona like that of the ember discharge under a sprite. We qualitatively explain the stepwise propagation of the negative gigantic jet discharge by combining the pilot streamer propagation model of Gallimberti et al.[6] with the electrostatic models of Raizer et al.[7,8] and Da Silva and Pasko[9] in which the density of the atmosphere decreases with altitude. In the latter models, the original leader potential drops across the streamer zone according to the internal electric field $E_{cr}$ scaling with the ratio of atmospheric density at that altitude compared to sea level ($\rho_0$ = 1.225 kg m$^{-3}$). We use the MSIS-E-90 atmospheric model[13] for more realistic profiles of air density ($\rho_z$) instead of a fixed scaling height of 7.2 km which we found to deviate significantly.

Like those previous models[6–9], we consider the (average) potential drop with distance across the streamer zone from a leader tip. This is the streamer stability field (or critical field), about 4.5 kV cm$^{-1}$ for positive streamers ($E_{cr+}$) and 7.5 to 12.5 kV cm$^{-1}$ ($E_{cr-}$) for negative streamers at sea level, scaling with density ($N_{amb}/N_0$, in our case $\rho_z/\rho_0$). The length of the streamer zone ahead of the leader tip reaches up to the distance where the potential at the streamer tip approaches the ambient potential. The aforementioned models considered that the streamer zone would need a high enough potential for a given leader tip altitude as to reach the ionosphere in one large step. However, the observed multiple steps suggest that the negative streamer corona does not jump directly from 20 km to the ionosphere. We now apply the concept described by Gallimberti et al.[6] (their Fig. 14) to the case of decreasing atmospheric density.

Explained graphically in **Supplementary Fig. 12a**, the discharge starts as intracloud lightning exiting the cloud (1). From the leader tip, the potential within the negative streamer zone drops with height according to their average stability field scaled by local atmospheric density (2). A relaxation process (3) starts as the streamer corona front approaches zero potential difference with the environment, adjusting its potential gradient toward the critical field of positive streamer corona (4). This increases the potential gradient between the front of the tip and the ionosphere again (not shown), so that a new bidirectional “pilot” discharge (5) can launch negative streamers upward, starting the next step. Notice how positive retrograde streamers are observed in the ember event (**Supplementary Figures 10 and 11**) and in laboratory negative streamer discharges in front of a leader tip (Les

Renardières Group[14]). These serve to bring more electrons upward to the space stem, increasing its potential needed for the next jump. This is similar to a process in positive streamers responsible for eventual upward negative streamer initiation in carrot sprites[15].

By experimentation (e.g. a Monte Carlo approach randomly varying the parameters) a solution can be obtained where the altitudes of the 3 lowest observed steps in GJ3 are matched (**Supplementary Fig. 12b**). This is the case for a leader tip at 18.6 (21.6) km with a potential of 277 (174) MV, in 8 (7) steps, when using $E_{cr-}$ of 12.5 kV cm$^{-1}$ and $E_{cr+}$ of 4.5 kV cm$^{-1}$ ($N_{amb}/N_0$). In fact, the fit required a fourth step starting from about 50 km altitude, which appears to correspond to the fork at (4) in **Fig. 2a** (main article). It must be noted that minimal changes in the $E_{cr}$ values, leader tip altitude or potential shift the step altitudes dramatically. As in previous electrostatic works[7–9], the temporal evolution of the streamer zone and its delays is not considered here, as this needs a dynamical model with chemistry. The leader height is assumed to be stationary, which may be the case between two steps. Also the potential of the leader tip is assumed to remain constant.

Note that the leader at 18.6 km is hidden by the cloud, and in the frame after the FDJ in GJ 3 (**Figure 1b,** main article), the leader has appeared reaching 21.7 km. This corresponds with one step and a propagation speed of $6 \times 10^4$ m s$^{-1}$, after which the propagation visually stops. This is compatible with the simulated speed and terminal altitudes by Da Silva and Pasko[16,17] for leaders with currents of 1-2 A (assuming a leader radius of 0.3 mm scaling with N0/N). However, their model assumed continuous leader propagation, while leader radius, which affects current density and heating as input for speed calculation, so far has never been optically confirmed at these altitudes, and was estimated by the authors to be higher in reality. Maintaining current density, this would increase the current.

Finally, an electric field can be assumed in front of the streamer tips, scaling inversely with the square of the distance from a spherical charge with the potential of the tip (**Supplementary Fig. 12c**). The charge is assumed to scale directly with the potential. This shows how initially the streamers may propagate given the thresholds at their tips are sufficient for propagation. With each step the breakdown threshold is reached more easily, as the geometry that dictates the field remains the same with altitude, while the reduction in atmospheric density lowers the requirements for breakdown and propagation. This may explain why the discharge no longer pauses above 40 km in GJ 3, increases in brightness and develops a large number of branches above 55 km as the field goes above the breakdown threshold and

no longer decreases, multiplying the free electrons. The acceleration at fields above $E_k$ is consistent with modeled streamers (e.g. [15]). In the studies of Raizer et al. and Da Silva and Pasko[7–9,16,17], acceleration was assumed to reflect the difference in propagation speed between leaders and streamers, while the latter[17] (figure 20b) actually show the acceleration of the jet to happen by a combination of the exponential upward extension of streamer corona and the leader tip moving upward. So, the increasing reduced electric field beyond the corona tips is an additional factor that controls the speed and the degree of branching, explaining the tree-like morphology of the gigantic jet.

Note that in real gigantic jets we do not expect the streamer zone to step at such regular intervals as this conceptual model, mainly because the potential drop over different streamer branches is not necessarily the same as the average $E_{cr-}$ value[18]. Furthermore, chemical processes which do not scale linearly with density may alter the conductivity and the resulting local potential gradient along the streamer zone.

An important implication of the observation of the jet likely being the streamer corona of a leader that stays around 20 km altitude (**Figure 1b** main article, **Supplementary Figure 5**) is that the cloud and leader voltages must be higher than often assumed: at least 150 MV. Similarly, it has recently been suggested (as one possible mechanism) that a leader must reach 100-350 MV to produce a terrestrial gamma-ray flash (TGF)[19–22]. In fact, it has recently been discovered by muon measurements[23] that some thunderstorms can apparently harbour potentials of 1300 MV. TGFs and gigantic jets both are very rare compared to regular lightning, with global estimates of 550,000 TGF[24] and 37,000 GJ per year[25] Extrapolating our campaign results, this number appears reasonable. It is in line with expectations that very few storms develop statistical outliers in charge configurations, among other possible requirements. Additional evidence for a large surplus of negative charge in the cloud and high leader potential are the negative sprites[26] that occurred just 2 minutes after GJ 4 and 18 minutes after GJ 12, but which appear to be still more common than gigantic jets themselves.

In summary, the electrostatic model updated with stepping fits the observed step altitudes in GJ 3, helping explain the morphological structure of the gigantic jet. The final jump is not only the last step toward the ionosphere, the large increase in voltage at the discharge front may exceed the breakdown threshold and explain the flaring out of multiple simultaneous streamers from that altitude. The cloud leader tip remaining at low altitude (around 20 km) is supported by **Fig. 1b** and **Supplementary Fig. 5** (as well as similar observations in works referenced in the main article). In such case, the leader

potential must reach values of several hundred MV, also to fit observed step sizes, which is higher than previously assumed.

# Supplementary References


1. Lee, L.-J., et al. Characteristics and generation of secondary jets and secondary gigantic jets, *J. Geophys. Res.*, **117,** A06317, https://doi.org/10.1029/2011JA017443 (2012).

2. Marshall, R. A. & Inan U. S. Possible direct cloud-to-ionosphere current evidenced by sprite-initiated secondary TLEs. *Geophys. Res. Lett.* **34**, L05806, https://doi.org/10.1029/2006GL028511 (2007).

3. Heavner, M. J. Optical spectroscopic observations of sprites, blue jets, and elves: Inferred microphysical processes and their macrophysical implications. PhD thesis, Univ. of Alaska Fairbanks, Fairbanks (2000).

4. Moudry, D. R. The dynamics and morphology of sprites. PhD thesis, Univ. of Alaska Fairbanks, Fairbanks (2003).

5. Stenbaek-Nielsen, H. C., Haaland, R., McHarg, M. G., Hensley, B. A. & Kanmae, T. Sprite initiation altitude measured by triangulation. *J. Geophys. Res.* **115,** A00E12, https://doi.org/10.1029/2009JA014543 (2010).

6. Gallimberti, I., Bacchiega, G., Bondiou-Clergerie, A. & Lalande, P. Fundamental processes in long air gap discharges. *C. R. Phys.* **3,** 1335–1359, https://doi.org/10.1016/S1631-0705(02)01414-7 (2002).

7. Raizer, Y. P., Milikh, G. M. & Shneider, M. N. On the mechanism of blue jet formation and propagation. *Geophys. Res. Lett.*, **33,** L23801, https://doi.org/10.1029/2006GL027697 (2006).

8. Raizer, Y. P., Milikh, G. M. & Shneider, M. N. Leader streamers nature of blue jets. *J. Atmos. Sol. Terr. Phys.* **69,** 925, https://doi.org/10.1016/j.Jastp.2007.02.007 (2007).

9. da Silva, C. L. & Pasko, V. P. Vertical structuring of gigantic jets. *Geophys. Res. Lett.* **40,** 3315–3319, https://doi.org/10.1002/grl.50596 (2013a).

10. Soula, S. et al. Gigantic jets produced by an isolated tropical thunderstorm near Réunion Island. *J. Geophys. Res.* **116,** D19103, https://doi.org/10.1029/2010JD015581 (2011).

11. Liu, N. et al. Upward electrical discharges observed above Tropical Depression Dorian. *Nat. Commun.* **6,** 5995 https://doi.org/10.1038/ncomms6995 (2015).

12. Liu, N. et al. High-altitude electrical discharges associated with thunderstorms and lightning. *J. Atmos. Sol. Terr. Phys.* http://dx.doi.org/10.1016/j.jastp.2015.05.013 (2015).

13. Hedin, A. E. Extension of the MSIS thermospheric model into the middle and lower atmosphere. *J. Geophys. Res.* **96,** 1159 (1991)

14. Les Renardières Group. Negative discharges in long air gaps at Les Renardières – 1978 results. *Electra* **74** (1981).

15. Luque, A. & Ebert, U. Sprites in varying air density: Charge conservation, glowing negative trails and changing velocity. *Geophys. Res. Lett.* **37,** L06806, https://doi.org/10.1029/2009GL041982 (2010).

16. da Silva, C. L. & Pasko, V. P. Simulation of leader speeds at gigantic jet altitudes. *Geophys. Res. Lett.* **39,** L13805, https://doi.org/10.1029/2012GL052251 (2012).

17. da Silva, C. L. & Pasko, V. P. Dynamics of streamer-to-leader transition at reduced air densities and its implications for propagation of lightning leaders and gigantic jets. *J. Geophys. Res.* **118,** 13561–13590 (2013b).

18. Luque A. & Ebert, U. Growing discharge trees with self-consistent charge transport: the collective dynamics of streamers. *New J. Phys.* **16,** 013039, https://doi.org/10.1088/1367-2630/16/1/013039 (2014).

19. Xu, W., Celestin, S. & Pasko, V. P. Source altitudes of terrestrial gamma-ray flashes produced by lightning leaders. *Geophys. Res. Lett.* **39,** L08801 (2012).

20. Celestin, S., Xu, W. & Pasko, V. P. Terrestrial gamma ray flashes with energies up to 100 MeV produced by nonequilibrium acceleration of electrons in lightning. *J. Geophys. Res.***117,** A05315, https://doi.org/10.1029/2012JA017535 (2012).

21. Mallios, S. A., Celestin, S. & Pasko, V. P. Production of very high potential differences by intracloud lightning discharges in connection with terrestrial gamma ray flashes. *J. Geophys. Res. Space Physics* **118,** 912–918, https://doi.org/10.1002/jgra.50109 (2013).

22. Skeltved, A. B., Østgaard, N., Mezentsev, A., Lehtinen, N. & Carlson, B. Constraints to do realistic modeling of the electric field ahead of the tip of a lightning leader. *J. Geophys. Res. Atmos.* **122,** 8120–8134, https://doi.org/10.1002/2016JD026206 (2017).

23. Hariharan, B., et al. Measurement of the electrical properties of a thundercloud through muon imaging by the GRAPES-3 experiment. *Phys. Rev. Lett.* **122,** 105101 (2019).

24. Østgaard, N., Albrecthsen, K. H., Gjesteland, T. & Collier A. A new population of terrestrial gamma-ray flashes in the RHESSI data. *Geophys. Res. Lett.* **42,** https://doi.org/10.1002/2015GL067064 (2015).

25. Chern, J. S., Wu, A. M. & Lin, S. F. Globalization extension of transient luminous events from FORMOSAT-2 observation. *Acta Astronaut.* **98,** 64–70, https://doi.org/10.1016/j.actaastro.2014.01.014 (2014).

26. Boggs, L. D. et al. An analysis of five negative sprite-parent discharges and their associated thunderstorm charge structures. *J. Geophys. Res. Atmos.* **121,** 759–784, https://doi.org/10.1002/2015JD024188 (2016).
