# Supplementary material for: Gigantic jet discharges evolve stepwise through the middle atmosphere
Source: Nat Commun. 2019 Sep 25;10:4350. doi: 10.1038/s41467-019-12261-y (PMC6761152; doi:10.1038/s41467-019-12261-y)
Supplement: Supplementary file 3 — Description of Additional Supplementary Files [file 41467_2019_12261_MOESM3_ESM.pdf]

Description of Additional Supplementary Files

File name: Supplementary Movie 1

Description: Compilation of all gigantic jets recorded by the detection camera in 2017 and 2018 in northern Colombia.

File name: Supplementary Movie 2

Description: Four gigantic jets as recorded by the high-speed cameras at 900 and 5000 images per second, in 2017 and 2018 respectively.
